# Information Deficits, Information Needs, and Preferences Regarding eHealth in a Dutch Population With Metabolic Dysfunction–Associated Steatotic Liver Disease: Cross-Sectional Survey Study

**DOI:** 10.2196/78536

**Published:** 2025-10-29

**Authors:** Sharon Oude Veldhuis, Maureen M J Guichelaar, Marjolein E M den Ouden, Lisette J E W C van Gemert-Pijnen, Constance H C Drossaert

**Affiliations:** 1 Research Group Technology, Health & Care, Saxion University of Applied Sciences, M.H. Tromplaan 28, Enschede, 7513 AB, The Netherlands, 088-0198888; 2 Centre for eHealth and Wellbeing Research, Section of Psychology, Health and Technology, University of Twente, Enschede, The Netherlands; 3 Department of Gastroenterology and Hepatology, Medisch Spectrum Twente Hospital, Enschede, The Netherlands; 4 Research Group Care and Technology, Regional Community College of Twente, Hengelo, The Netherlands

**Keywords:** metabolic dysfunction–associated steatotic liver disease, MASLD, information provision, information needs, patient education, needs assessment, eHealth

## Abstract

**Background:**

Globally, metabolic dysfunction–associated steatotic liver disease (MASLD) is a common lifestyle-related disease. Lifestyle interventions focusing on healthy eating habits, physical exercise, and reducing body weight in case of obesity are the primary recommended therapies to reverse or improve MASLD. However, patients often experience difficulties in complying with the required lifestyle changes for several reasons, including a lack of knowledge. Health care professionals express limited time during consultations as one of the barriers to discussing lifestyle behavior change. A potential solution to eliminate these barriers and improve the provision of information to patients with MASLD is the use of eHealth.

**Objective:**

This study aimed to explore the information needs and deficits of patients with MASLD regarding a variety of disease-related topics and their preferences regarding a future eHealth intervention.

**Methods:**

In a cross-sectional survey study, patients with MASLD were recruited via 2 Dutch patient organizations. The questionnaire included questions on sociodemographics, information provision and needs, and preferences regarding an eHealth intervention. Data were reported using descriptive statistics. Pearson chi-square tests and logistic regression analysis were used to identify differences in outcomes between subgroups.

**Results:**

The questionnaire was filled out by 449 respondents (women: 363/449, 81%; age: mean 56, SD 11 y). Fewer than 20% of them indicated that they had received sufficient information on a broad range of disease-related topics. Approximately 72% (325/449) to 90% (405/449) of respondents indicated that they would like to receive additional information. Respondents who did not know their disease stage reported a significantly higher need for information on general topics, compared to respondents who reported their disease stage (*P* values ranging from <.01 to .03). Respondents with (self-reported) metabolic dysfunction–associated steatohepatitis were more interested in contact with fellow patients than respondents with an early or unknown stage of disease (*P*=.002). Regarding a future eHealth intervention, respondents were most interested in receiving MASLD-related information, practical examples, and references to relevant websites or apps. Respondents were least interested in contact, collaboration, or competition with other app users.

**Conclusions:**

The vast majority of respondents reported a high rate of information deficits on a broad range of MASLD-related topics and expressed a strong need for additional information. Insights into information needs and preferences regarding eHealth can be used to develop an eHealth intervention for patients with MASLD.

## Introduction

### Background

Globally, metabolic dysfunction–associated steatotic liver disease (MASLD) is a common lifestyle-related disease. MASLD encompasses a spectrum of diseases, ranging from simple steatosis (accumulation of fat in the liver cells) to metabolic dysfunction–associated steatohepatitis (MASH; liver inflammation) and liver cirrhosis (scarring of the liver as a result of long-standing inflammation). This latter stage of disease increases the risk of hepatocellular carcinoma (HCC) and liver failure and is associated with increased rates of liver transplantation and liver-related mortality [1,2]. Furthermore, MASLD is associated with increased rates of cardiovascular disease [3,4]. The current global prevalence of MASLD is estimated to be 32% [5], and it is expected to rise because of the ongoing obesity epidemic and the rise of people with type 2 diabetes mellitus [6]. Moreover, MASLD is expected to emerge as the main cause of end-stage liver disease in the coming decades [5,7]. Lifestyle interventions focusing on healthy eating habits (Mediterranean or low-carb diet), physical exercise, and reducing body weight in case of obesity are the primary recommended therapies to reverse or improve MASLD. However, previous studies showed that changing lifestyles and complying with the required lifestyle changes are difficult for several reasons, including difficulties with incorporating lifestyle changes, motivation, and a lack of social support [8,9].

From the patients’ perspective, previous studies showed that many patients with MASLD experienced an information deficit [10-12]. However, an overview of topics on which patients would like to receive more information is lacking. Moreover, it is unclear if the information needs are associated with patient characteristics, such as MASLD stage, time since diagnosis, education level, age, and gender. This poor provision of MASLD-related information can result in reduced knowledge about disease- and treatment-related topics [8,10,12] and, therefore, less concern and awareness of the serious adverse outcomes related to MASLD [8] and difficulties with disease acceptance due to misconceptions [13]. Health care professionals play an important role in the provision of information. A previous qualitative study showed that limited time during consultations is one of the barriers to discussing lifestyle behavior change [12]. In addition, a lack of expertise regarding MASLD among health care professionals, particularly in primary care [10,12], could hinder adequate information provision. Providing patients with correct information can increase their self-efficacy [13]. A higher self-efficacy and better illness perception were key determinants of successful lifestyle changes in patients with MASLD [14]. One of the potential solutions to eliminate these barriers and improve information provision to patients with MASLD is the use of eHealth. eHealth offers the opportunity to disseminate information to a broad public, with minimal effort from health care professionals. Previous studies have shown that eHealth can lead to a better quality of care, quality of life, and clinical outcomes, as it increases self-care and contributes to better communication between patients and health care providers [15-17]. A recent systematic review and meta-analysis of MASLD-related studies reported a significant effect of eHealth interventions on the decrease of BMI and liver enzymes [18]. These eHealth interventions were delivered via different channels (eg, telephone, mobile app, and web-based platform) and consisted of a variety of components, such as information, goal setting, counseling, reminders, and feedback.

In general, tailoring eHealth interventions to the needs and wishes of the target population is recommended. Possible ways to improve effectiveness and adherence to eHealth include integrating persuasive system design (PSD) principles [19], such as tailoring and competition, and behavior change techniques (BCTs) [20], such as goal setting and just-in-time coaching [21,22]. These techniques and design features may also be supportive for patients with MASLD. However, insights into the preferences of patients with MASLD or specific subgroups regarding persuasive eHealth features and BCTs are lacking.

### Objectives

This study aimed to explore information needs and deficits in a large patient population with MASLD regarding a variety of disease-related topics. Furthermore, associations between information needs and patient characteristics were examined, as well as eHealth preferences. These insights are essential for improving MASLD-related information provision and empowering patients to adopt positive lifestyle changes.

## Methods

### Study Design

A cross-sectional online survey was shared via social media of the Dutch Digestive Foundation and the Dutch Liver Patients Association. The Dutch Digestive Foundation and Dutch Liver Patients Association are nationwide patient organizations for patients with gastrointestinal and liver-related diseases as well as their relatives. The organizations strive to optimize the quality of care and quality of life for these patient groups.

### Respondents and Procedures

Patients were invited to participate in the online survey if they were diagnosed with MASLD and were aged ≥18 years. No exclusion criteria were applicable. The online questionnaire was open for response from June 1, 2021, to September 12, 2021.

The STROBE (Strengthening the Reporting of Observational Studies in Epidemiology) checklist for cross-sectional studies was used for writing this paper (Checklist 1).

### Ethical Considerations

The study protocol was approved by the institutional review board of Medisch Spectrum Twente (K21-16). All respondents were informed about the scope and aim of the study via an online patient information letter. They provided online informed consent before enrollment in the study. Respondents did not receive any compensation for taking part in the study. No directly identifiable patient data were collected, and all data were securely stored on a server at Saxion University of Applied Sciences.

### Questionnaire

#### Sociodemographic and Health-Related Variables

Sociodemographic characteristics that were assessed included age, gender (woman, man, and nonbinary), educational level (primary, lower secondary, upper secondary, bachelor’s, master’s, and other), type of mobile devices used (smartphone, computer, tablet, and wearables), and daily use of the internet (yes or no).

Regarding health-related variables, we asked when and by whom MASLD was diagnosed (gastroenterologist, general practitioner, nurse practitioner, and other) and the (self-reported) stage of the disease (simple steatosis, MASH without fibrosis or cirrhosis, MASH with fibrosis or cirrhosis, MASLD and HCC, unknown: has not been told, unknown: it has not been examined, and cannot remember but it has been told).

Education levels were merged into 3 subgroups—basic (primary and lower secondary), intermediate (upper secondary), and advanced (bachelor’s and master’s)—based on the International Standard Classification of Education [23], to reduce the number of subgroups.

#### Information Provision and Needs

Before the survey, 6 interviews were performed with patients with MASLD about their illness perceptions and barriers and motivators for lifestyle change. Relevant topics from these interviews were incorporated in the survey. The survey included questions about information provision and needs. First, the respondents were asked to give their opinion about the quantity of received information on a variety of MASLD-related topics (what is MASLD, causes, consequences, treatment, self-management, how to improve the eating and exercise pattern, contact with other patients with MASLD, support by health care professionals, and recommended websites and apps). Response options were no information, little information, and sufficient information. Second, respondents were asked if they would have liked to receive more information on these topics (yes or no). Third, they were asked by whom the MASLD-related information was provided (gastroenterologist or hepatologist, general practitioner, nurse practitioner, dietician, physiotherapist, and other) and what sources they used when looking for more information. Finally, we identified patients’ experiences with information provision and their suggestions for improvement of information provision using an open-ended question.

#### Preferences Regarding an eHealth Intervention for Patients With MASLD

Preferences of patients with MASLD regarding a future eHealth intervention were explored using a selection of 15 potential features (as shown in the *Preferences Regarding an eHealth Intervention for Patients With MASLD* section in *Results*) derived from the PSD principles [19] and BCTs (derived from the Theoretical Domains Framework) [20]. The selection of features is based on the target group and the aim of a future eHealth intervention (improving disease-related knowledge and supporting lifestyle change). The following features were incorporated: self-monitoring, rewards, reminders, competition, social learning, cooperation, social support, praise, goal setting, information about the disease, feedback on behavior, reduction, tunneling, suggestions, and instructions. The formulation of these principles and techniques was simplified in the questionnaire to ensure understanding by respondents. For each item, respondents were asked to what extent it appealed to them (appeals a lot, appeals a little, and does not appeal). Furthermore, suggestions for the development of a future eHealth intervention were asked using an open-ended question.

### Statistical Analysis

For the questions on the quantity of information received and eHealth preferences, answering options were coded as 0=no information or does not appeal, 1=little information or appeals a little, and 2=sufficient information or appeals a lot; and a mean score was calculated per item. The missing data were avoided by configuring all questionnaire items as forced responses.

Descriptive statistics were reported as means with SDs or medians with IQRs for continuous variables, and numbers with percentages for categorical data. Pearson chi-square tests were used to examine whether there were differences in information needs and preferences regarding eHealth features between subgroups based on gender, education, and MASLD stage. Logistic regression analysis was used to examine the association between information needs and the continuous variables age and time since diagnosis. Significance levels were set at α=.05 for all tests.

Quantitative data were analyzed using SPSS Statistics (version 26.0; IBM Corp). Data from open-ended questions were coded and categorized in Atlas.ti (version 23.0; Scientific Software Development GmbH) by 2 researchers (SOV and MEMDO).

## Results

### Characteristics of Study Respondents

In total, 1242 respondents started the questionnaire. A total of 469 respondents completed at least 34% of the questionnaire, including the questions on sociodemographics, health-related topics, and information provision and needs. The questions about eHealth features were filled out by 366 respondents; others dropped out due to the questionnaire’s length. In total, 20 respondents were excluded based on their questionnaire comments (eg, aged <18 y or not having an official diagnosis). Hence, results on information provision and eHealth features were described for 449 and 348 respondents, respectively. The sociodemographic and health-related characteristics of these groups did not differ substantially.

The majority of respondents were women (363/449, 81%), with a mean age of 56 (SD 11; range 25-83) years, as reported in Table 1. Most respondents (193/449, 43%) had completed education at an intermediate level. Almost all respondents (326/348, 94%) used (mobile) devices and the internet on a daily basis. Simple steatosis (206/449, 46%) was the most common self-reported stage of MASLD among respondents. Interestingly, more than one-third of the respondents (161/449, 36%) stated that they did not know their stage of disease. The time since diagnosis of MASLD ranged from 2 to 46 years, with a median of 5 (IQR 5) years. More than half of the respondents (271/449, 60%) mentioned that the gastroenterologist diagnosed MASLD.

**Table 1.** Sociodemographic and health-related characteristics (N=449).

Characteristics	Value
Age (years), mean (SD)	56 (11)
Gender, n (%)	
Woman	363 (81)
Man	85 (19)
Nonbinary	1 (0)
Education	
Basic	124 (28)
Intermediate	193 (43)
Advanced	129 (29)
Other (not specified)	3 (1)
Use of devices^a^, n (%)	
Smartphone	329 (95)
Computer	287 (83)
Tablet	216 (62)
Wearables (eg, smartwatch)	83 (24)
Daily use of internet^a^, n (%)	326 (94)
Self-reported stage of MASLD^b^, n (%)	
Simple steatosis	206 (46)
MASH^c^ without fibrosis or cirrhosis	29 (7)
MASH with fibrosis or cirrhosis	46 (10)
MASLD and hepatocellular carcinoma	2 (0.4)
Unknown: has not been told	88 (20)
Unknown: has not been examined	62 (14)
Cannot remember, but it has been told	11 (2)
Other	5 (1)
Time since diagnosis of MASLD (years), median (IQR)	5 (5)
Diagnostician, n (%)	
Gastroenterologist	271 (60)
General practitioner	96 (21)
Nurse practitioner	18 (4)
Other	64 (14)

a Variables “use of devices” and “daily use of internet”; n=348.

b MASLD: metabolic dysfunction–associated steatotic liver disease.

c MASH: metabolic dysfunction–associated steatohepatitis.

### Information Provision and Needs

Most respondents indicated that they had received no or only little information on a variety of MASLD-related topics (Table 2). The topics with the highest percentages of sufficient information were “causes of MASLD” and “consequences of MASLD,” but these percentages were still only 18% (81/449 and 82/449, respectively). The lowest percentages of sufficient information were reported for “recommended websites or apps“ and “contact with other patients with MASLD” (23/449, 5% and 12/449, 3%, respectively). For almost all disease-related and health care–related topics, 72% (325/449) to 90% (405/449) of respondents indicated a need to receive additional information. Only 37% (165/449) of respondents expressed a need for more information on the topic “opportunities to get in contact with other patients with MASLD.”

**Table 2.** Amount of information received and information needs regarding different metabolic dysfunction–associated steatotic liver disease (MASLD)–related topics (N=449).

Topics	No information (score=0), n (%)	Little information (score=1), n (%)	Sufficient information (score=2), n (%)	Score (0‐2), mean	Need for more information, n (%)
Treatment of MASLD	225 (50.1)	171 (38.1)	53 (11.8)	0.6	405 (90.2)
Self-management	211 (47.0)	173 (38.5)	65 (14.5)	0.7	403 (89.8)
Causes of MASLD	178 (39.6)	190 (42.3)	81 (18.0)	0.8	394 (87.8)
Consequences of MASLD	213 (47.4)	154 (34.3)	82 (18.3)	0.7	392 (87.3)
What is MASLD	168 (37.4)	202 (45.0)	79 (17.6)	0.8	382 (85.1)
How to improve diet	217 (48.3)	158 (35.2)	74 (16.5)	0.7	377 (84.0)
Recommended websites or apps	343 (76.4)	83 (18.5)	23 (5.1)	0.3	336 (74.8)
How to improve exercise pattern	224 (49.9)	154 (34.3)	71 (15.8)	0.7	333 (74.2)
Support by health care professionals	303 (67.5)	109 (24.3)	37 (8.2)	0.4	325 (72.4)
Contact with other patients with MASLD	376 (83.7)	61 (13.6)	12 (2.7)	0.2	165 (36.7)

### Providers of Information Regarding MASLD

Almost 47% (210/449) of the respondents mentioned that they had received information about their disease from the gastroenterologist or hepatologist. Others stated that they had been informed by the general practitioner (123/449, 27%), nurse practitioner (45/449, 10%), dietician (44/449, 10%), physiotherapist (5/449, 1%), or other (42/449, 9%). Approximately 20% (95/449) of the respondents indicated that they had not received information about MASLD from a health care provider.

### Searching for Additional Information About MASLD

In addition to the information provided by health care professionals, 78% (349/449) of the respondents mentioned that they had searched for additional information about MASLD via different channels. The internet was the most used source to search for information (302/449, 67%). In total, 35% (156/449) of the respondents searched on disease-specific websites (eg, the website of the patient organization). Some other channels were used by only a few respondents: information from acquaintances (13/449, 3%), fellow patients (7/449, 2%), dieticians (5/449, 1%), books (3/449, 1%), other physicians or medical specialists (3/449, 1%), patient organizations (2/449, <1%), and scientific papers (1/449, <1%).

### Associations Between Information Needs and Patient Characteristics

Regarding self-reported stage of disease, significant differences were found between the 3 subgroups on 6 of the 10 topics on information needs (Table 3). Patients with MASH (with or without fibrosis, cirrhosis, or HCC) expressed a significantly higher need for more information on the topic “Contact with other patients with MASLD” and a significantly lower need to receive information on the topic “How to improve diet,” compared to respondents with an early stage (simple steatosis) or unknown stage of disease. Respondents who did not know their stage of disease reported a significantly higher need for more information on the topic “What is MASLD,” compared to respondents who did report their stage of disease (Table 3). They also reported a significantly higher need for more information on the topics “self-management,” “causes of MASLD,” and “consequences of MASLD,” compared to respondents with MASH (with or without fibrosis, cirrhosis, or HCC). When looking at gender, women expressed, overall, a somewhat higher need for information compared to men (Table 3). Their information needs were significantly higher on the topics “What is MASLD,” “Treatment of MASLD,” “Consequences of MASLD,” and “How to improve diet.” Information needs of respondents did not differ significantly by education level, age, or time since diagnosis.

**Table 3.** Differences in information needs by (self-reported) metabolic dysfunction–associated steatotic liver disease (MASLD) stage and gender.

Need for more information (yes)	MASLD stage (n=444^a^)				Gender (n=448^b^)		
	Simple steatosis (n=206), n (%)	MASH^c^ with or without fibrosis, cirrhosis, or HCC^d^ (n=77), n (%)	Stage unknown by respondent (n=161), n (%)	*P* value	Woman (n=363), n (%)	Man (n=85), n (%)	*P* value
Treatment of MASLD	187 (90.8)	66 (85.7)	147 (91.3)	.36	333 (91.7)	72 (84.7)	.048
Self-management	186 (90.3)	63 (81.8)	150 (93.2)^e^	.02	331 (91.2)	72 (84.7)	.07
Causes of MASLD	182 (88.3)	61 (79.2)	147 (91.3)^e^	.03	323 (89.0)	70 (82.4)	.09
Consequences of MASLD	180 (87.4)	61 (79.2)	147 (91.3)^e^	.03	324 (89.3)	67 (78.8)	.01
What is MASLD	171 (83.0)	58 (75.3)	148 (91.9)^f^	.002	320 (88.2)	61 (71.8)	<.001
How to improve diet	176 (85.4)	56 (72.7)^g^	141 (87.6)	.01	314 (86.5)	63 (74.1)	.01
Recommended websites or apps	153 (74.3)	61 (79.2)	119 (73.9)	.64	275 (75.8)	60 (70.6)	.32
Support by health care professional	152 (73.8)	52 (67.5)	118 (73.3)	.56	262 (72.2)	62 (72.9)	.89
How to improve exercise pattern	149 (72.3)	54 (70.1)	127 (78.9)	.24	276 (76.0)	57 (67.1)	.09
Contact with other patients with MASLD	68 (33.0)	42 (54.5)^g^	54 (33.5)	.002	132 (36.4)	33 (38.8)	.67

a Subgroup with MASLD stage “other*” *(n=5) was excluded from this analysis.

b Nonbinary (n=1) was excluded from this analysis.

c MASH: metabolic dysfunction–associated steatohepatitis.

d HCC: hepatocellular carcinoma.

e Significantly different from subgroup “MASH (with or without fibrosis, cirrhosis, or HCC)”; *P*<.05).

f Significantly different from subgroups “simple steatosis” and “MASH (with or without fibrosis, cirrhosis, or HCC)”; *P*<.01).

g Significantly different from subgroups “simple steatosis” and “stage unknown by respondent” (*P*≤.01).

### Suggestions for Improving Information Provision

Respondents were asked, via an open-ended question, what could be improved in the provision of MASLD-related information. This open question was answered by 221 respondents. Several respondents (22/221, 10%) mentioned that information provision in total should be improved. Furthermore, many respondents (56/221, 25%) answered that they would have liked the information provided to be clearer or more in-depth. Other respondents made this more specific by mentioning that the information could be improved regarding the causes (18/221, 8%), prevention (6/221, 3%), consequences (14/221, 6%), prognosis (4/221, 2%), lifestyle advice and self-management (41/221, 19%), the relation with other diseases and medication (7/221, 3%), and possibilities of support by other health care providers (4/221, 2%). Some respondents (5/221, 2%) explicitly suggested improving the availability of reliable and relevant information about MASLD:

A clear leaflet or website with information on what can possibly be done about it [MASLD]. I was told by the doctor’s assistant that a slightly fatty liver is not a serious illness and that a large proportion of people have it without knowing it. I received no advice on what I could possibly do about it.

In addition, respondents (37/221, 17%) mentioned that health care professionals could be more proactive in providing information about MASLD, rather than merely communicating the diagnosis. A few respondents (9/221, 4%) indicated that they felt that health care professionals themselves were not well informed about MASLD in general and how it should be managed. Other respondents (16/221, 7%) mentioned that they felt the risks of MASLD were trivialized, for example, by saying that nothing needs to be done about it:

That gastroenterologists should receive more information to inform patients.

Others (2/221, 1%) reported that health care professionals made them feel guilty that they were diagnosed with MASLD, which they experienced as unpleasant. Furthermore, some respondents (5/221, 2%) would have appreciated more medical tests and follow-up:

Don’t make it feel like it’s your own fault.

More frequent monitoring when improvement is in progress. This motivates to continue on the path taken.

A number of respondents (8/221, 4%) indicated that they believed that there should be more nationwide awareness regarding MASLD. Some respondents (23/221, 10%) expressed specific preferences for the format of information provision, such as a brochure, website, app, information sessions, or a special improvement program for MASLD (comparable to those for cardiac rehabilitation). Only a few respondents (7/221, 3%) indicated that information provision did not need to be improved.

### Preferences Regarding an eHealth Intervention for Patients With MASLD

Regarding a future eHealth intervention, most respondents (329/348, 95%) mentioned that they were interested in receiving information about MASLD, as well as practical examples and references to relevant websites or apps (both 323/348, 93%) (Table 4). Respondents were least interested in eHealth interventions that include contact with other patients with MASLD (168/348, 48%), collaboration with other app users (164/348, 47%), or competition (93/348, 27%). Subgroup analysis regarding the eHealth features showed that patients with MASH (with or without fibrosis, cirrhosis, or HCC) preferred the options “competition between app users” (*P*=.004), “learning from experiences of others” (*P*=.002), and “possibility to contact other patients with MASLD” (*P*<.001) significantly more often than patients with an early (simple steatosis) or unknown stage of MASLD. No other significant associations were found between the eHealth preferences and the stage of disease. In the open-ended question, 23% (51/221) of the respondents elaborated on this topic with additional suggestions for a future eHealth intervention. These suggestions included push messages, free availability of the app, ease of use, and interoperability with other (eHealth) devices. Furthermore, patients indicated that eHealth interventions can help them recognize their (unhealthy) lifestyle patterns and highlighted the value of regular reminders for making lifestyle changes. However, the intervention should also take into account that patients have multimorbidity. Finally, some respondents preferred personal contact with professionals.

**Table 4.** Preferences regarding a future eHealth intervention (n=348).

Items for an eHealth intervention (based on PSD^a^ principles and BCTs^b^)	Does not appeal (score=0), n (%)	Appeals a little (score=1), n (%)	Appeals a lot (score=2), n (%)	Score (0‐2), mean
Receiving information about MASLD^c^	19 (5.5)	108 (31.0)	221 (63.5)	1.6
Practical examples (eg, recipes)	25 (7.2)	122 (35.1)	201 (57.8)	1.5
References to relevant websites or apps	25 (7.2)	156 (44.8)	167 (48.0)	1.4
Goal setting	56 (16.1)	149 (42.8)	143 (41.1)	1.3
Self-monitoring	79 (22.7)	123 (35.3)	146 (42.0)	1.2
Feedback on progress	74 (21.3)	143 (41.1)	131 (37.6)	1.2
Testing knowledge (eg, quiz)	76 (21.8)	155 (44.5)	117 (33.6)	1.1
Possibility to contact health care professionals	64 (18.4)	180 (51.7)	104 (29.9)	1.1
Compliments in case of progress	100 (28.7)	116 (33.3)	132 (37.9)	1.1
Reminders	106 (30.5)	128 (36.8)	114 (32.8)	1.0
Learning from experiences of others	98 (28.2)	179 (51.4)	71 (20.4)	0.9
Getting rewards for progress	138 (39.7)	118 (33.9)	92 (26.4)	0.9
Possibility to contact other patients with MASLD	180 (51.7)	112 (32.2)	56 (16.1)	0.6
Collaboration with app users	184 (52.9)	108 (31.0)	56 (16.1)	0.6
Competition between app users	255 (73.3)	63 (18.1)	30 (8.6)	0.4

a PSD: persuasive system design.

b BCT: behavior change technique.

c MASLD: metabolic dysfunction–associated steatotic liver disease.

## Discussion

### Principal Findings

MASLD is a lifestyle-related disease, and lifestyle improvements are the mainstay in therapy as well as the prevention of disease progression. However, previous studies have shown that limited knowledge about MASLD and the recommended lifestyle changes can hinder effective lifestyle improvements. [8,12]. Our study showed that less than 20% of patients with MASLD indicated that they received sufficient information about a broad range of disease-related topics. In addition, more than one-third of our study respondents (161/449) indicated that they do not know their stage of disease. Respondents who did not know the severity of their disease stage reported a significantly higher need for information on general MASLD-related topics, compared to respondents who reported their stage of disease. Respondents who reported to have MASH (with and without fibrosis, cirrhosis, or HCC) expressed a significantly higher need for information on the topic “Contact with other patients with MASLD.” According to respondents, information provision can be improved by more detailed information (eg, causes, prevention, consequences, and prognosis) and the availability of reliable and relevant information about MASLD. In addition, respondents mentioned that health care professionals could be more proactive in providing information about MASLD, rather than simply communicating the diagnosis. There were also some specific wishes mentioned by respondents regarding the form of information provision, such as a brochure, website, or app. Almost 80% (349/449) of respondents sought additional information about their disease, with the internet being the most frequently used source. Despite their additional search for disease-related information, 72% (325/449) to 90% (405/449) of respondents reported that they would like to receive additional information on almost all disease-related topics, including MASLD in general, self-management, and treatment of MASLD. This may indicate that it is difficult for patients to find the specific disease-related information they requested or that they do not know where to find the right information. Both topics were addressed by several respondents in our survey.

Regarding an eHealth intervention, respondents were most interested in receiving MASLD-related information, practical examples, and references to relevant websites or apps. Respondents were least interested in contact, collaboration, or competition with other app users. However, in line with the results on information needs, patients with MASH (with or without fibrosis, cirrhosis, or HCC) were significantly more interested in features related to contact with other patients with MASLD than patients with an early or an unknown stage of MASLD. This may be explained by the experience of more disease symptoms, concerns, and a higher burden on daily life. Hence, these patients might have a greater need for recognition, emotional support, and sharing experiences with fellow patients, compared to patients with simple steatosis who, generally, experience fewer physical and mental complaints [24]. Our findings are consistent with the study of Valery et al [25], which showed that patients with a good to moderate impaired hepatic function (Child-Pugh A and B) had higher information needs compared to patients with advanced hepatic dysfunction (Child-Pugh C). Moreover, patients with a Child-Pugh C score had higher practical and physical needs, as well as psychosocial issues [25]. These findings suggest that it is important for health care professionals to be aware of potential differences in information needs between patients, as tailored patient education is important for prolonged lifestyle changes [8].

Previous studies, mostly qualitative [10-12,26], have identified several reasons for the lack of information among patients with MASLD. These reasons included time limitations during consultations [12], as well as low motivation of health care professionals to invest their time during consultation in educating patients with MASLD because they experienced that patients often did not follow their advice (ie, not worthwhile to invest time in lifestyle support) [12]. Health care professionals also reported that they lacked adequate knowledge to inform patients [10,27]. The latter was also supported by our study, in which a few respondents mentioned that they felt that their health care professional lacked the disease-specific knowledge to educate them about MASLD and the required lifestyle changes.

Given the difficulties of changing lifestyle among patients with MASLD, a first step toward better lifestyle support is improving information provision in patients. The development and implementation of an eHealth intervention for patients with MASLD is one of the solutions. An eHealth intervention can save time during consultations and complement the motivation and knowledge level of health care professionals. This study gave a first impression of preferences of patients with MASLD regarding a future eHealth intervention, based on a selection of PSD principles and BCTs. To our knowledge, no previous studies were conducted on the preferences of patients with MASLD regarding eHealth. However, a previous qualitative study of people with obesity [21] showed that this comparable study population had similar preferences regarding PSD principles and BCTs, such as the need for reliable information and suggestions about healthy eating. In addition to eHealth interventions, future studies should also focus on the role and education of health care professionals regarding MASLD to enhance information provision for patients.

Although improved knowledge about MASLD and related lifestyle changes is expected to greatly contribute to lifestyle change in patients with MASLD, a future eHealth intervention should not be limited to the provision of information, practical examples, and references to other relevant websites or apps. To achieve and maintain sustainable lifestyle change, behavior change models show that it is also important to strengthen internal motivation and pay attention to patients’ physical and social environment [28,29]. For example, these factors may be addressed by incorporating options for setting personal goals, enabling self-monitoring, and making the eHealth app accessible to patients’ relatives to enhance social support.

As the study was conducted during the COVID-19 pandemic, it is plausible that patients with MASLD are now more familiar with digital health than they were at the time of data collection. The field of digital care is evolving rapidly, and large language models have been widespread and adopted in recent years [30,31]. However, preferences of patients with MASLD regarding the eHealth features are not expected to have changed substantially, as they were mainly related to obtaining reliable information about MASLD. As not all individuals possess the digital competencies required to retrieve reliable information using large language models, it is likely that the need for a tailored eHealth intervention for patients with MASLD, including reliable information, remains high.

### Strengths and Limitations

The strengths of the study include the large sample size of patients with MASLD (with different severity levels) and an overview of the information needs of Dutch patients with MASLD, which was not studied before. In addition, the study provides relevant insights into eHealth preferences, which is relevant for future eHealth interventions to make it suitable for its target population. Limitations of the study include the self-reported status of the MASLD stages. Hence, the results of the subgroup analysis regarding the disease stage should be interpreted with some caution. Moreover, our study showed that information needs were significantly higher among women compared to men. This was also found in a previous study among patients recovering from an acute ischemic coronary event [32]. Given the overrepresentation of women in our study (which was also found in previous online surveys) [33], the information needs in this study might be somewhat higher compared to those in the patient population, in which the prevalence of MASLD is higher among men than women [34]. Although the exact reasons behind these skewed distributions remain unclear [33], one possible explanation for the overrepresentation of women in this study is that the recruitment took place via the social media of 2 patient organizations. Women generally engage in online health information–seeking behavior more frequently than men and are also more likely to use health forums [35,36]. In contrast*,* the results emphasize the necessity to tailor information provision to the needs of each individual patient. In addition, further research is necessary to identify the preferences of patients with MASLD regarding PSD principles and BCTs, as these entities were briefly addressed. Therefore, these results can be best seen as an indication, instead of a complete overview of preferences for developing a future eHealth intervention.

### Conclusions

In conclusion, our study supports that inadequate patient education limits the daily practice of MASLD care. The vast majority of respondents (approximately 80%) perceived no or limited education about their disease and disease-related entities, including self-management. Although almost all respondents undertook additional searches for information, the majority of respondents would like to receive more information about a broad array of MASLD-related topics. Patient education is of utmost importance in the daily practice of MASLD care, as the causes as well as treatment of the disease are lifestyle related. This study contributes to a better understanding of how patients with MASLD experience the current information provision and emphasizes the importance of improving information provision for a broad range of topics. In addition, it supports a necessity to develop eHealth interventions for patients with MASLD and their health care professionals, as eHealth can be used to provide reliable information and reduce the impact of disparities in MASLD-related knowledge between health care professionals. The initial insights from patients with MASLD regarding their eHealth preferences, as provided by this study, may serve as a valuable starting point for developing such an eHealth intervention.

## Supplementary material

10.2196/78536 Checklist 1 STROBE checklist for cross-sectional studies.

## Abbreviations

BCT: behavior change technique
HCC: hepatocellular carcinoma
MASH: metabolic dysfunction–associated steatohepatitis
MASLD: metabolic dysfunction–associated steatotic liver disease
PSD: persuasive system design
STROBE: Strengthening the Reporting of Observational Studies in Epidemiology

## References

[1] Burra P, Becchetti C, Germani G (2020). NAFLD and liver transplantation: disease burden, current management and future challenges. JHEP Rep.

[2] Mattos ÂZ, Debes JD, Dhanasekaran R (2021). Hepatocellular carcinoma in nonalcoholic fatty liver disease: a growing challenge. World J Hepatol.

[3] Kasper P, Martin A, Lang S (2021). NAFLD and cardiovascular diseases: a clinical review. Clin Res Cardiol.

[4] Duell PB, Welty FK, Miller M (2022). Nonalcoholic fatty liver disease and cardiovascular risk: a scientific statement from the American Heart Association. Arterioscler Thromb Vasc Biol.

[5] Teng ML, Ng CH, Huang DQ (2023). Global incidence and prevalence of nonalcoholic fatty liver disease. Clin Mol Hepatol.

[6] Younossi ZM, Koenig AB, Abdelatif D, Fazel Y, Henry L, Wymer M (2016). Global epidemiology of nonalcoholic fatty liver disease-meta-analytic assessment of prevalence, incidence, and outcomes. Hepatology.

[7] Riazi K, Azhari H, Charette JH (2022). The prevalence and incidence of NAFLD worldwide: a systematic review and meta-analysis. Lancet Gastroenterol Hepatol.

[8] Tincopa MA, Wong J, Fetters M, Lok AS (2021). Patient disease knowledge, attitudes and behaviours related to non-alcoholic fatty liver disease: a qualitative study. BMJ Open Gastroenterol.

[9] Heredia NI, Thrift AP, Balakrishnan M (2022). Perceived barriers to weight loss among Hispanic patients with non-alcoholic fatty liver disease. Hisp Health Care Int.

[10] Avery L, Exley C, McPherson S, Trenell MI, Anstee QM, Hallsworth K (2017). Lifestyle behavior change in patients with nonalcoholic fatty liver disease: a qualitative study of clinical practice. Clin Gastroenterol Hepatol.

[11] Haigh L, Bremner S, Houghton D (2019). Barriers and facilitators to Mediterranean diet adoption by patients with nonalcoholic fatty liver disease in Northern Europe. Clin Gastroenterol Hepatol.

[12] Hallsworth K, Dombrowski SU, McPherson S, Anstee QM, Avery L (2020). Using the theoretical domains framework to identify barriers and enabling factors to implementation of guidance for the diagnosis and management of nonalcoholic fatty liver disease: a qualitative study. Transl Behav Med.

[13] Hallsworth K, Avery L, Trenell MI (2016). Targeting lifestyle behavior change in adults with NAFLD during a 20-min consultation: summary of the dietary and exercise literature. Curr Gastroenterol Rep.

[14] Zelber-Sagi S, Bord S, Dror-Lavi G (2017). Role of illness perception and self-efficacy in lifestyle modification among non-alcoholic fatty liver disease patients. World J Gastroenterol.

[15] Granja C, Janssen W, Johansen MA (2018). Factors determining the success and failure of eHealth interventions: systematic review of the literature. J Med Internet Res.

[16] Renzi E, Baccolini V, Migliara G (2022). The impact of eHealth interventions on the improvement of self-care in chronic patients: an overview of systematic reviews. Life (Basel).

[17] (2023). More care providers recognise added value of eHealth. National Institute for Public Health and the Environment.

[18] Zafar Y, Sohail MU, Saad M (2025). eHealth interventions and patients with metabolic dysfunction-associated steatotic liver disease: a systematic review and meta-analysis. BMJ Open Gastroenterol.

[19] Oinas-Kukkonen H, Harjumaa M (2009). Persuasive systems design: key issues, process model, and system features. Commun Assoc Inf Syst.

[20] Michie S, Richardson M, Johnston M (2013). The behavior change technique taxonomy (v1) of 93 hierarchically clustered techniques: building an international consensus for the reporting of behavior change interventions. Ann Behav Med.

[21] Asbjørnsen RA, Wentzel J, Smedsrød ML (2020). Identifying persuasive design principles and behavior change techniques supporting end user values and needs in eHealth interventions for long-term weight loss maintenance: qualitative study. J Med Internet Res.

[22] Gemert-Pijnen L, Kelders SM, Beerlage-de Jong N, Oinas-Kukkonen H, van Gemert-Pijnen L, Kelders SM, Kip H, Sanderman R (2018). eHealth Research, Theory and Development: A Multi-Disciplinary Approach.

[23] International Standard Classification of Education (ISCED). International Labour Organization.

[24] Younossi Z, Aggarwal P, Shrestha I (2022). The burden of non-alcoholic steatohepatitis: a systematic review of health-related quality of life and patient-reported outcomes. JHEP Rep.

[25] Valery PC, Bernardes CM, Mckillen B (2021). The patient’s perspective in cirrhosis: unmet supportive care needs differ by disease severity, etiology, and age. Hepatol Commun.

[26] Cook NS, Nagar SH, Jain A (2019). Understanding patient preferences and unmet needs in non-alcoholic steatohepatitis (NASH): insights from a qualitative online bulletin board study. Adv Ther.

[27] Driessen S, de Jong VD, van Son KC (2023). A global survey of health care workers’ awareness of non-alcoholic fatty liver disease: the AwareNASH survey. United European Gastroenterol J.

[28] Michie S, van Stralen MM, West R (2011). The behaviour change wheel: a new method for characterising and designing behaviour change interventions. Implement Sci.

[29] Prochaska JO, DiClemente CC (1983). Stages and processes of self-change of smoking: toward an integrative model of change. J Consult Clin Psychol.

[30] Cuff A (2023). The evolution of digital health and its continuing challenges. BMC Digit Health.

[31] Maity S, Saikia MJ (2025). Large language models in healthcare and medical applications: a review. Bioengineering (Basel).

[32] Stewart DE, Abbey SE, Shnek ZM, Irvine J, Grace SL (2004). Gender differences in health information needs and decisional preferences in patients recovering from an acute ischemic coronary event. Psychosom Med.

[33] Becker R (2022). Gender and survey participation: an event history analysis of the gender effects of survey participation in a probability-based multi-wave panel study with a sequential mixed-mode design. Methods Data Anal.

[34] Feng G, Targher G, Byrne CD (2025). Global burden of metabolic dysfunction-associated steatotic liver disease, 2010 to 2021. JHEP Reports.

[35] Baumann E, Czerwinski F, Reifegerste D (2017). Gender-specific determinants and patterns of online health information seeking: results from a representative German health survey. J Med Internet Res.

[36] Bidmon S, Terlutter R (2015). Gender differences in searching for health information on the internet and the virtual patient-physician relationship in Germany: exploratory results on how men and women differ and why. J Med Internet Res.

